# Supplementary material for: Insights into the mechanism of oligodendrocyte protection and remyelination enhancement by the integrated stress response
Source: bioRxiv. 2023 Jan 23:2023.01.23.525156. Preprint. [Version 1] doi: 10.1101/2023.01.23.525156 (PMC9900777; doi:10.1101/2023.01.23.525156)

bioRxiv preprint doi: https://doi.org/10.1101/2023.01.23.525156; this version posted January 23, 2023. The copyright holder for this preprint (which was not certified by peer review) is the author/funder, who has granted bioRxiv a license to display the preprint in perpetuity. It is made available under aCC-BY 4.0 International license.

Figure 1-figure supplement 1

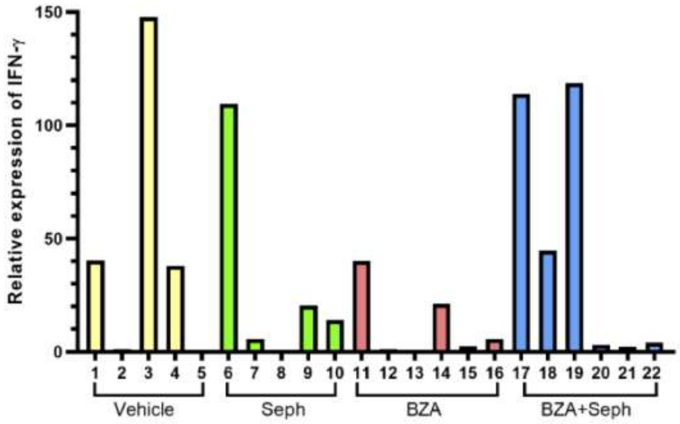

bioRxiv preprint doi: https://doi.org/10.1101/2023.01.23.525156; this version posted January 23, 2023. The copyright holder for this preprint (which was not certified by peer review) is the author/funder, who has granted bioRxiv a license to display the preprint in perpetuity. It is made available under aCC-BY 4.0 International license.

Figure 3-figure supplement 2

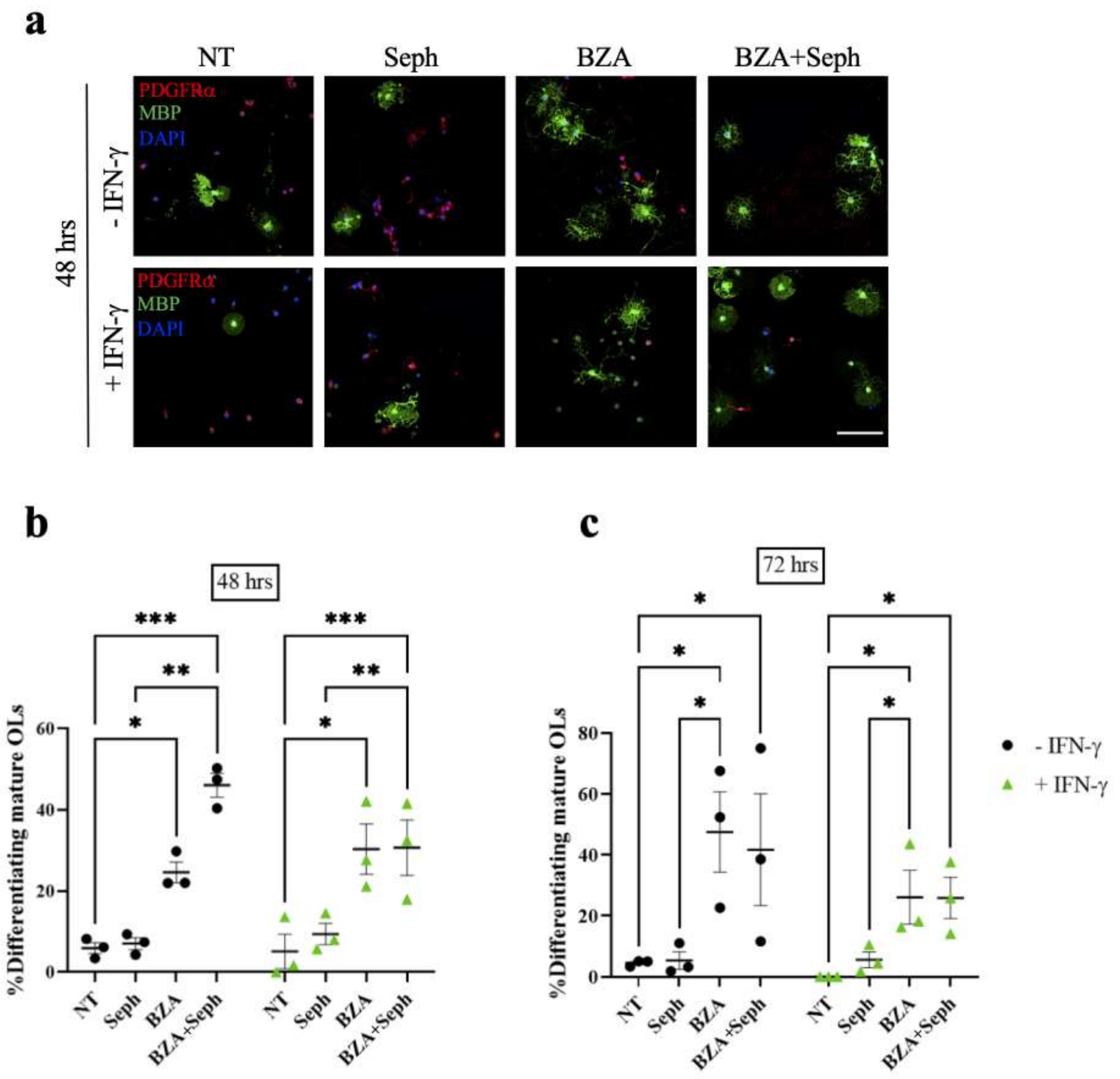

bioRxiv preprint doi: https://doi.org/10.1101/2023.01.23.525156; this version posted January 23, 2023. The copyright holder for this preprint (which was not certified by peer review) is the author/funder, who has granted bioRxiv a license to display the preprint in perpetuity. It is made available under aCC-BY 4.0 International license.

Figure 5-figure supplement 3

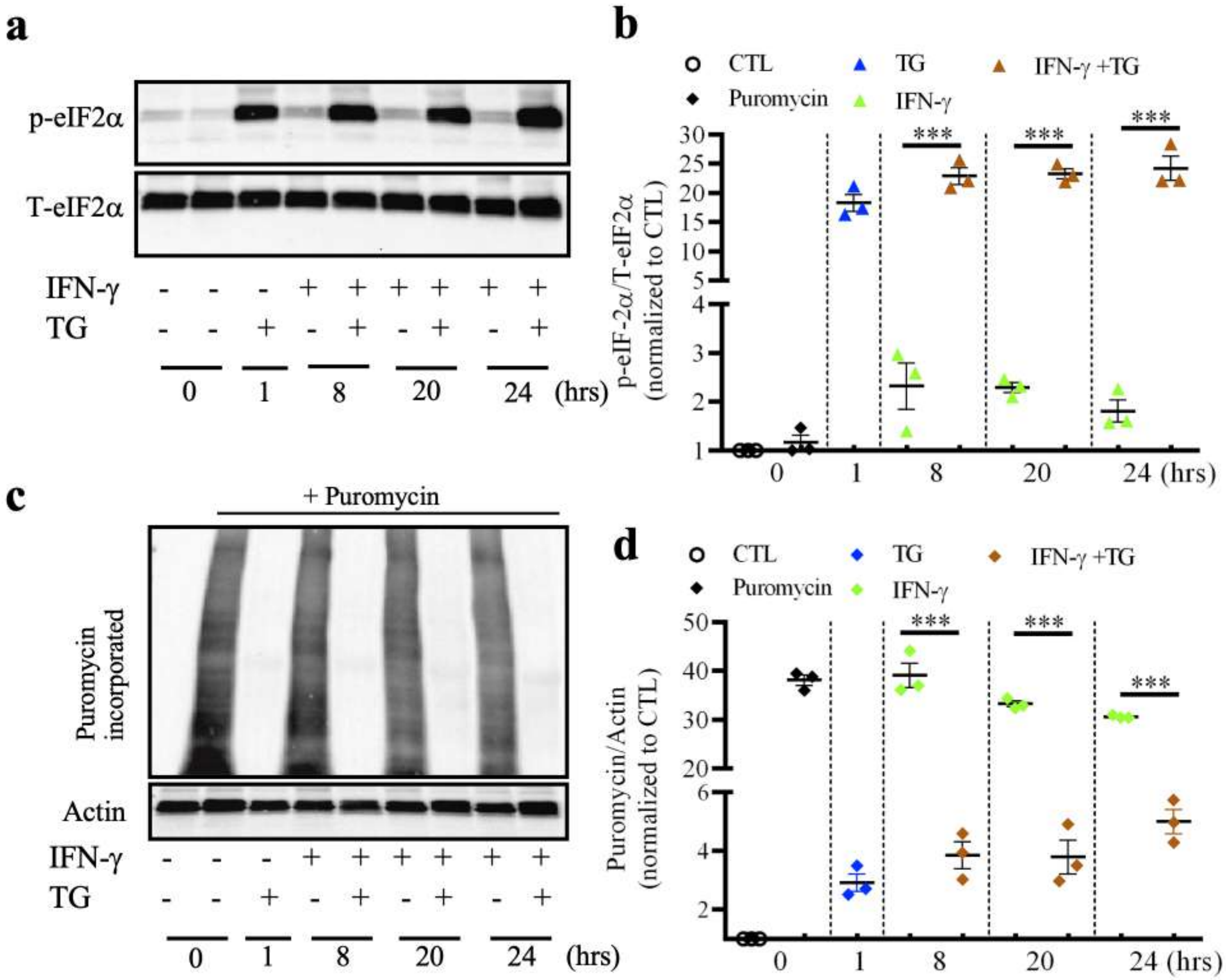

bioRxiv preprint doi: https://doi.org/10.1101/2023.01.23.525156; this version posted January 23, 2023. The copyright holder for this preprint (which was not certified by peer review) is the author/funder, who has granted bioRxiv a license to display the preprint in perpetuity. It is made available under aCC-BY 4.0 International license.

Figure 6-figure supplement 4

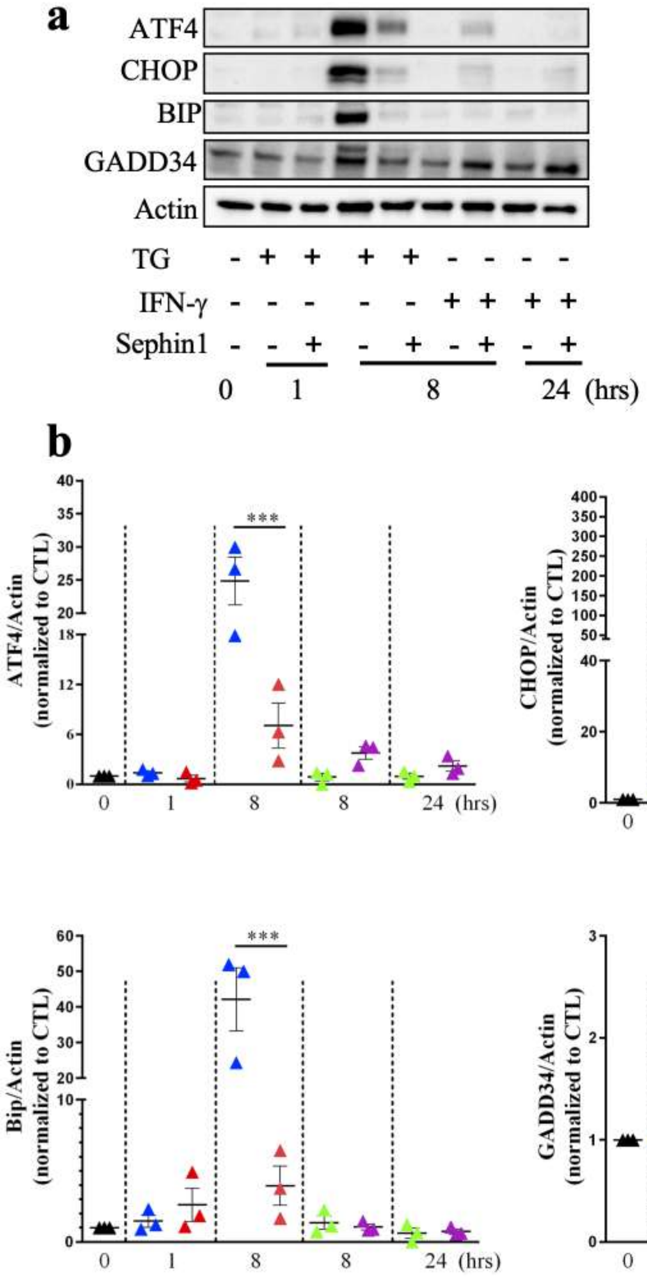

bioRxiv preprint doi: https://doi.org/10.1101/2023.01.23.525156; this version posted January 23, 2023. The copyright holder for this preprint (which was not certified by peer review) is the author/funder, who has granted bioRxiv a license to display the preprint in perpetuity. It is made available under aCC-BY 4.0 International license.

Figure 7-figure supplement 5

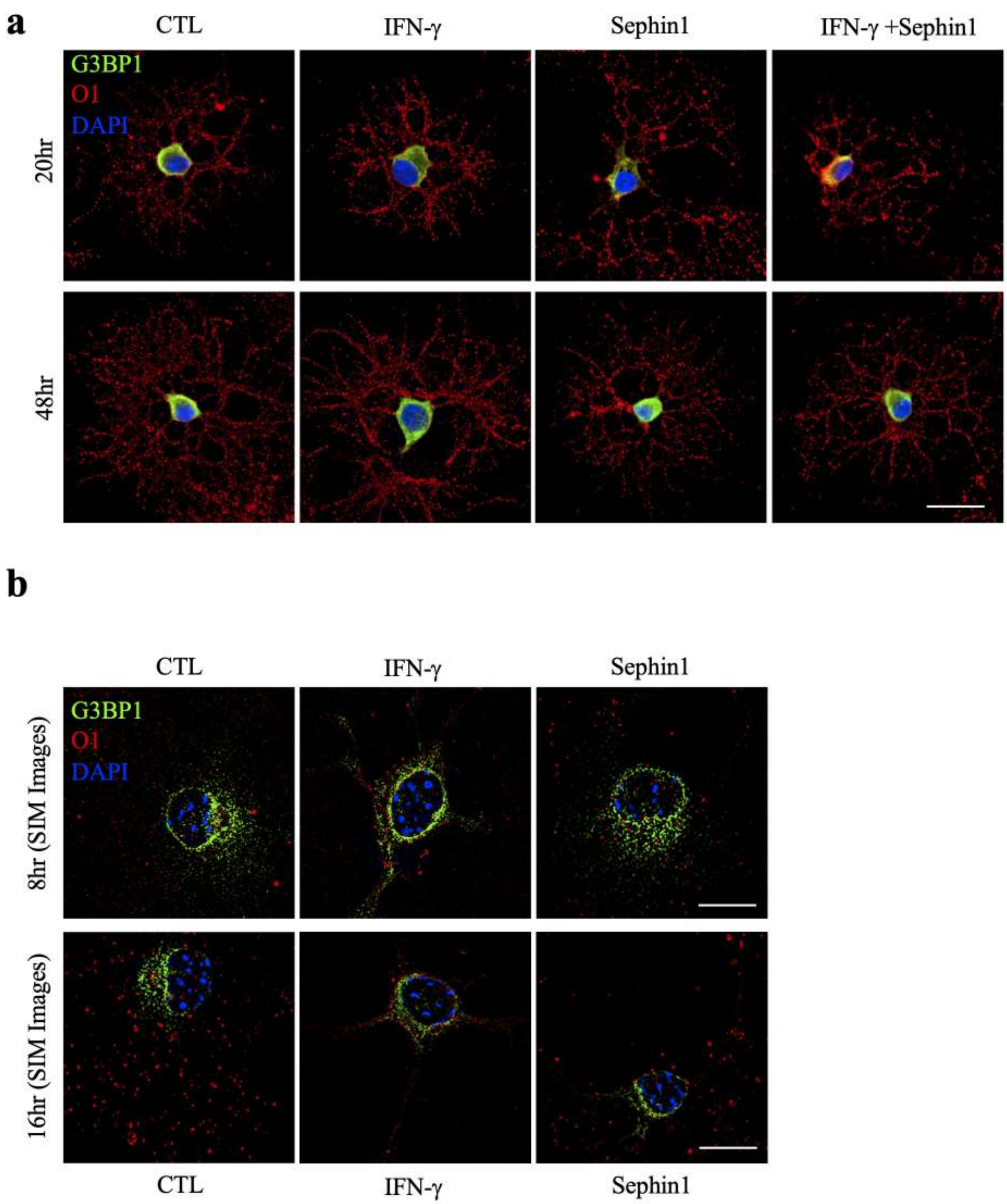

Supplement: Supplement 1 — Figure 1-figure supplement 1. GFAP-tTA;TRE-IFN-γ mice express IFN-γ after release from doxycycline. The expression levels of IFN-γ in the cerebellum two weeks after cuprizone withdrawal in each treatment group (RT-PCR). Each bar is an individual mouse. Mouse #1, 3, 4, 6, 7, 9, 10, 11, 14, 16, 17, 18 and 19 were selected for further examination. Figure 3-figure supplement 2. Combined treatment of Sephin1 and BZA promote OPC differentiation at 48 and 72 hours. (a) PDGFR-α and MBP immunostaining of OPCs in cultures that were seeded for 48 hours. Cells exposed to IFN-γ (+ IFN-γ) were treated with non-treatment (NT), Sephin1 (Seph1), BZA, or BZA plus Seph1. Cells not exposed to IFN-γ (−IFN-γ) were used as controls. Scale bar: 100µm. Quantification of percentage of cells positive for MBP (differentiating oligodendrocytes) over the total number of oligodendrocyte lineage cells at 48 hours (b) and 72 hours (c). *p < 0.05, **p < 0.01. Figure 5-figure supplement 3. IFN-γ combined with TG is able to induce the ISR and reduces the overall protein translation in mouse oligodendrocytes. (a) Western blot of oligodendrocytes treated by IFN-γ and/or TG. (b) Quantification of a Western blot for p-eIF2α levels. (c) Western blot of puromycin incorporation assay using an anti-puromycin antibody. OPCs were differentiated in differentiation media overnight and then treated with IFN-γ and/or TG. Puromycin was added 30 min before harvesting for puromycin labeling. (d) Quantification of Western blot. Data are mean ± SEM from three biological isolations and technique replicates. ***p < 0.001. Figure 5-figure supplement 3-source data 1. Western-blots images of p-eIF2α and puriomycin in oligodendrocytes exposed to IFN-γ and/or Thapsigargin (TG). Figure 6-figure supplement 4. Sephin1 increases the expression of ISR downstream targets. Immunoblot analysis of p-eIF2α, total eIF2α and ISR response (ATF4, BIP, GADD34, and CHOP) in either TG or IFN-γ exposed developing oligodendrocytes. Cells were tr [file NIHPP2023.01.23.525156v1-supplement-1.pdf]
